# Fibrous dysplasia of the jaws: four cases with conservative and resective surgical management

**DOI:** 10.1080/23320885.2026.2646776

**Published:** 2026-03-22

**Authors:** Nandika Desta Dewara, Bramasto Purbo Sejati, Erdananda Haryosuwandito

**Affiliations:** a Resident of Oral and Maxillofacial Surgery Study Program, Faculty of Dentistry, Universitas Gadjah Mada, Yogyakarta, Indonesia; b Department of Oral and Maxillofacial Surgery, Faculty of Dentistry, Universitas Gadjah Mada, Yogyakarta, Indonesia

**Keywords:** Bone reshaping, fibro-osseous tumor, fibrous dysplasia, resective surgery

## Abstract

**Introduction:**

Fibrous dysplasia (FD) is a slow-growing, benign fibro-osseous tumor with a recurrent character distinguished by replacement with fibrous connective tissue instead of normal bone. The medullary bone is substituted by fibrous connective tissue, leading to immature, poorly mineralized bone. FD is responsible for approximately 2.5% of all bone tumors and 7% of all benign bone tumours. Patients may show involvement of one bone (monostotic FD; MFD) or multiple bones (polyostotic FD; PFD). The present case report describes FD’s clinical, imaging and therapeutic aspects in the maxillary and mandibular region.

**Case:**

We present four cases of fibrous dysplasia affecting the maxilla and mandibular region, which were identified over 6 months at a single institution. All (*n* = 4) patients underwent surgical procedures: 2 patients in the mandibular region, one in the maxillary region, and one in the maxillary and mandibular region. No complications occurred at the six-month follow-up.

**Case management:**

The diagnosis of FD is established based on clinical, radiographic, and histopathological features. Imaging analysis showed an expansive, non-destructive mass, with a well-circumscribed cortical and appearance of ground glass, involving either the maxilla or mandible. After diagnosis of FD, 3 patients undergo a conservative bone reshaping surgical procedure, and 1 patient undergoes a surgical resection and bone reshaping with primary wound closure.

**Conclusion:**

The diagnosis of FD remains challenging because of its clinical, imaging, and histological similarities with other fibro-osseous tumors. A conservative approach of bone reshaping could be adopted as the first line of treatment for patients suffering from FD. A complete surgical removal by resective surgery and immediate reconstructive procedures also produces satisfactory clinical results after adequate follow-up.

## Introduction

The term of fibrous dysplasia was first mentioned by Lichtenstein, an American pathologist, in 1938 [1]. Fibrous dysplasia (FD) is a developmental bone disorder that originates from a genetic defect disturbing the osteogenesis process leading to the replacement of normal bone with the excess proliferation of fibrous tissue that undergoes abnormal mineralization [2,3]. It can be associated with hormonal dysregulations, most frequently precocious puberty with cutaneous manifestations seen in the McCune-Albright syndrome, MAS, or intramuscular myxomas in Mazabraud’s syndrome, MS [2]. Fibrous dysplasia is categorized as a rare bone disorder constituting 2.5% of all bone diseases and 7% of benign bone tumors, highlights its rarity within the spectrum of bone disorders [4]. Fibrous dysplasia typically presents as a slow-growing mass in the affected bone or bones. This mass can cause various symptoms, including pain, especially when it involves weight-bearing bones. It can also lead to facial asymmetry when it affects bones in the craniofacial region [5].

Fibrous dysplasia, based on the extent of bone involvement, is categorized into monostotic and polyostotic [6]. Monostotic fibrous dysplasia (MFD) is around 10 times more common and often presents unilaterally, affecting only one side of the body or one bone [1,2]. On imaging analysis, FD typically presents as an expansive, nondestructive mass, usually exhibiting a well-circumscribed cortical border and a characteristic ‘ground glass’ appearance [7]. Histologically, there are curved trabeculae of woven bone, typically having a hook-like form or resembling Chinese characters, entwined in a bland fibrous stroma devoid of any malignant cellular characteristics, and there is no osteoblastic rimming [6,7].

Fibrous dysplasia arises from a post-zygotic somatic mutation in the GNAS gene, which encodes the alpha subunit of the stimulatory G protein [6,8]. This mutation causes the GNAS1 gene in osteoblastic progenitor cells to become constitutively active, leading to continuous stimulation of adenylate cyclase [1,9]. As a result, cyclic adenosine monophosphate (cAMP) levels increase, promoting excessive cell proliferation and abnormal bone differentiation. Malignant transformation in fibrous dysplasia is rare, occurring in less than 1% of cases. Signs of potential malignancy include persistent pain, rapid enlargement, and elevated alkaline phosphatase levels [10].

This manuscript presents four cases of MFD affecting the maxillary and mandibular region, seen in patients across various age groups. One MFD affecting the maxilla was seen in a 62-year-old elderly female patient. Two MFD cases were found in the mandibular area, one seen in a young 4-year-old male child and the other in a 23-year-old adult female. The last case was found in the combined maxillary and mandibular region in a 15-year-old female patient. Different imaging modalities are used to establish the diagnosis, which is then confirmed by biopsy. The chosen course of treatment depends on each case, involving bone debulking, remodelling, and resection surgery to improve the patient’s function and aesthetic factors.

## Patients

Informed consent was obtained from all patients or guardians; the study was exempt from IRB review as a retrospective case series

### Case 1

#### Subjective and objective examination

**Figure 1.**
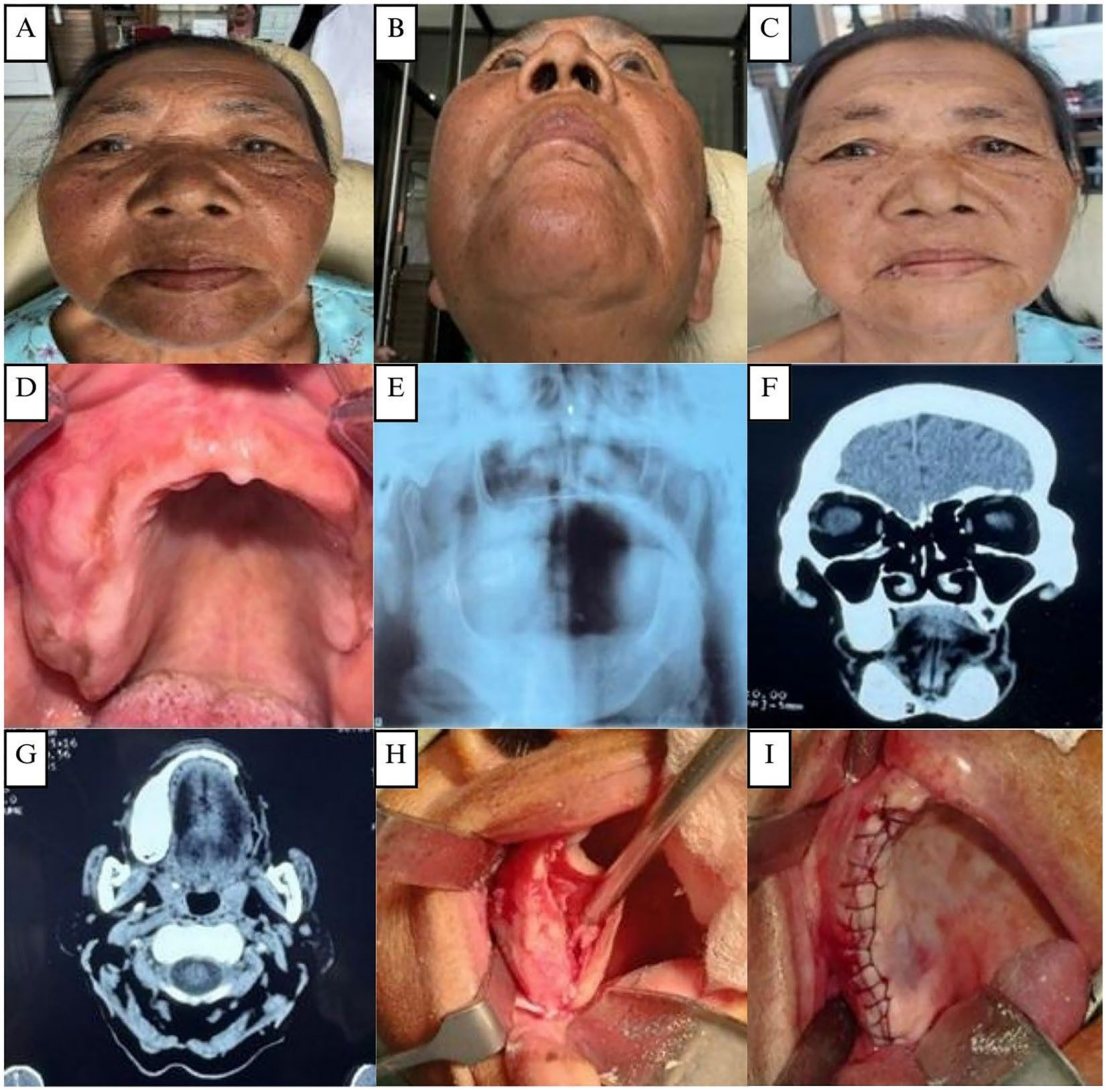
**(A and B)** The first case, a 62-year-old elderly female patient with swelling in the right maxillary area. **(C)** Clinical appearance 2 weeks post-operative. **(D)** Pre-operative intraoral clinical appearance **(E)**. OPG showing a fully edentulous area with no osteodestruction. **(F–G)** CT scan showing a hyperdense lesion in the right maxilla measuring 28x14 mm. **(H–I)** Surgical exposure, bone reshaping, and layers of closure of the right maxillary alveolar arch.

A 62-year-old elderly female was referred to Oral and Maxillofacial Clinics, RSUD Temanggung, with a chief complaint of swelling in the right posterior alveolar area (Figure 1(A and B)). She first noticed the swelling 8 years ago. She felt that it increased slowly but steadily until its present size. There was a history of incisional biopsy in 2018 with a diagnosis of fibrous dysplasia. The patient felt mild pain for the last 2 months. The patient had a medical history of hypertension, but no history of allergies.

On examination, there was an apparent facial asymmetry caused by a diffuse bony expansion of the posterior third of the right alveolar ridge around the 15–18 region, which extended to the maxillary tuberosity area (Figure 1(D)). The swelling has a smooth surface, bony hard on palpation, and a normal pinkish color like the surrounding gum area. Differential diagnoses included ossifying fibroma, myxoma, and osteosarcoma.

On orthopantomography (OPG), multiple missing teeth were found, no osteodestruction was found, and no clear signs of fibrous dysplasia were seen (Figure 1(E)). A CT scan revealed a hyperdense lesion measuring 28 × 14 mm in the right maxilla. No erosion was seen in the bone, no tumor was seen in the paranasal sinuses, and no clear intracerebral hyperdense lesion was seen (Figure 1(F and G)).

The patient underwent surgical excision and bone recontouring under general anesthesia. The bone swelling was exposed using an intraoral approach with a midline incision in the edentulous right maxilla. The tumor appeared pale tan with increased vascularity. Partial resection and osteoplasty were performed using fissure and carbide Fraser burs to smooth the bone margins (Figure 1(H)). The bone was brown, gritty, and softer than normal, with moderate bleeding. The site was closed in layers with 3/0 polysorb silk and a pressure dressing applied (Figure 1(I)). Postoperatively, the patient reported no pain and was satisfied with the aesthetic improvement of facial asymmetry (Figure 1(C)).

**Figure 2.**
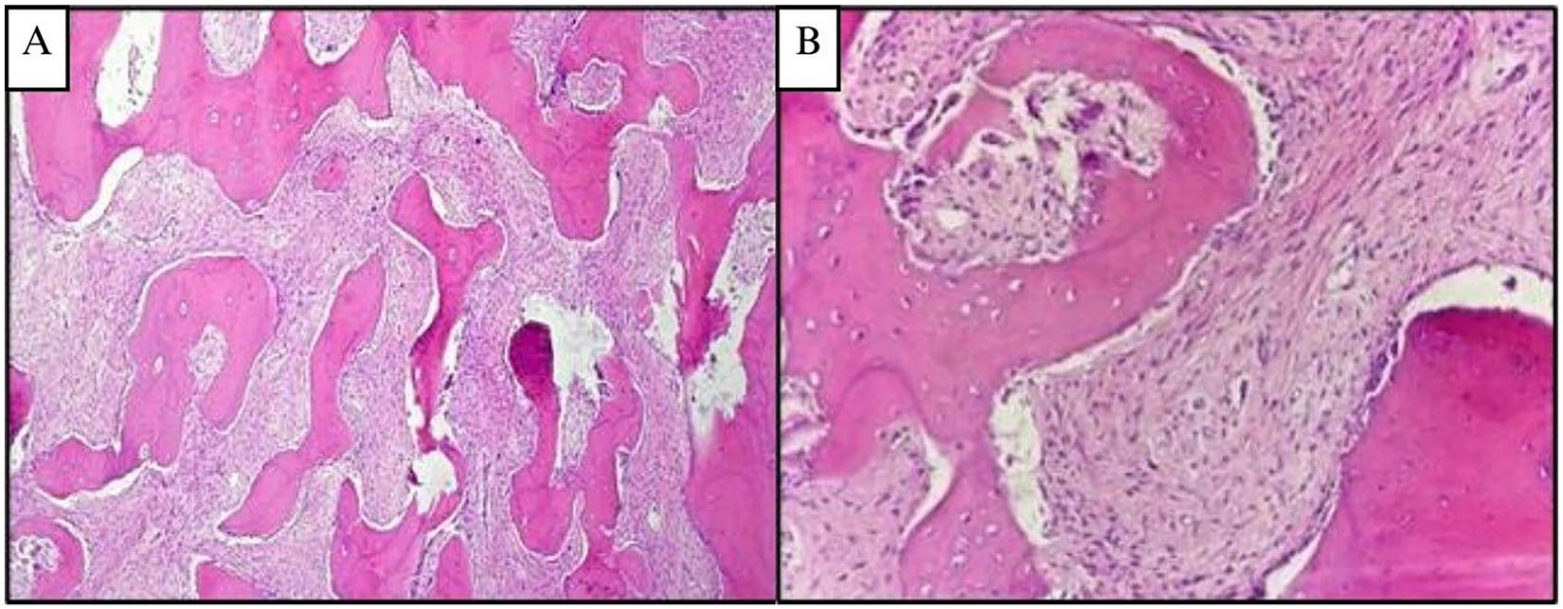
**(A and B) Case 1**, H&E sections at 4× and 20× magnification, showing delicate, branching, curvilinear trabeculae, C shape (‘fish hook configuration’) pattern of immature woven one exhibiting osteoblastic rimming, scattered in a dense and richly cellular fibrovascular connective tissue stroma.

The histopathological examination of the surgically removed sections from the mass showed bone trabecular tissue with various shapes, some showing a C shape (‘fish hook configuration’), accompanied by proliferation of fibrous connective tissue stromal cells. There were no signs of malignancy (Figure 2(A and B)). The conclusion was a fibrous dysplasia of the right maxilla.

### Case 2

#### Subjective and objective examination

**Figure 3.**
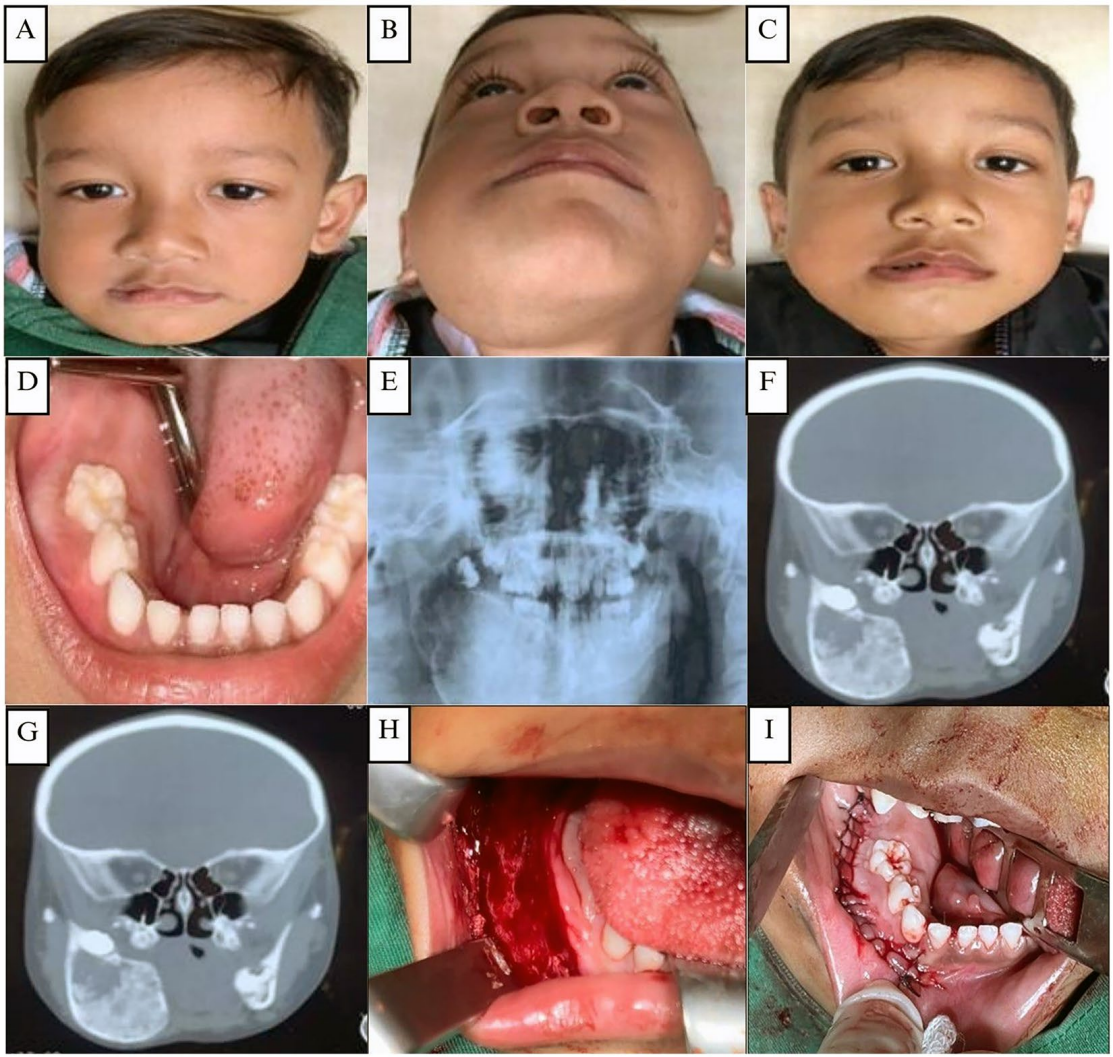
**(A and B)** The second case, a 4-year-old male child patient with swelling in the right mandibular area. **(C)** Clinical appearance 2 weeks post-operative. **(D)** Pre-operative intraoral clinical appearance **(E)**. OPG showing a radiolucent lesion in the right mandibular area with an amorphous shape with multiple unerupted permanent teeth. **(FG)** CT scan showing a hard tissue mass in the right mandible area with cystic lesions, expansile lesions with a size of 37.3 × 45.7 mm, oval shape with firm boundaries. **(H–I)** Right mandibular intraoral area: surgical exposure, bone reshaping, and layers of closure.

A 4-year-old male child presented to the Oral and Maxillofacial Clinics, RSUD Temanggung, with his parents complaining of swelling and asymmetry in the right side of his mandible (Figure 3(A and B)). Over the last 7 months, the swelling gradually increased, with no complaint of pain. There was a history of incisional biopsy in 2023 with a diagnosis of fibrous dysplasia. The patient had no history of allergies.

**Figure 4.**
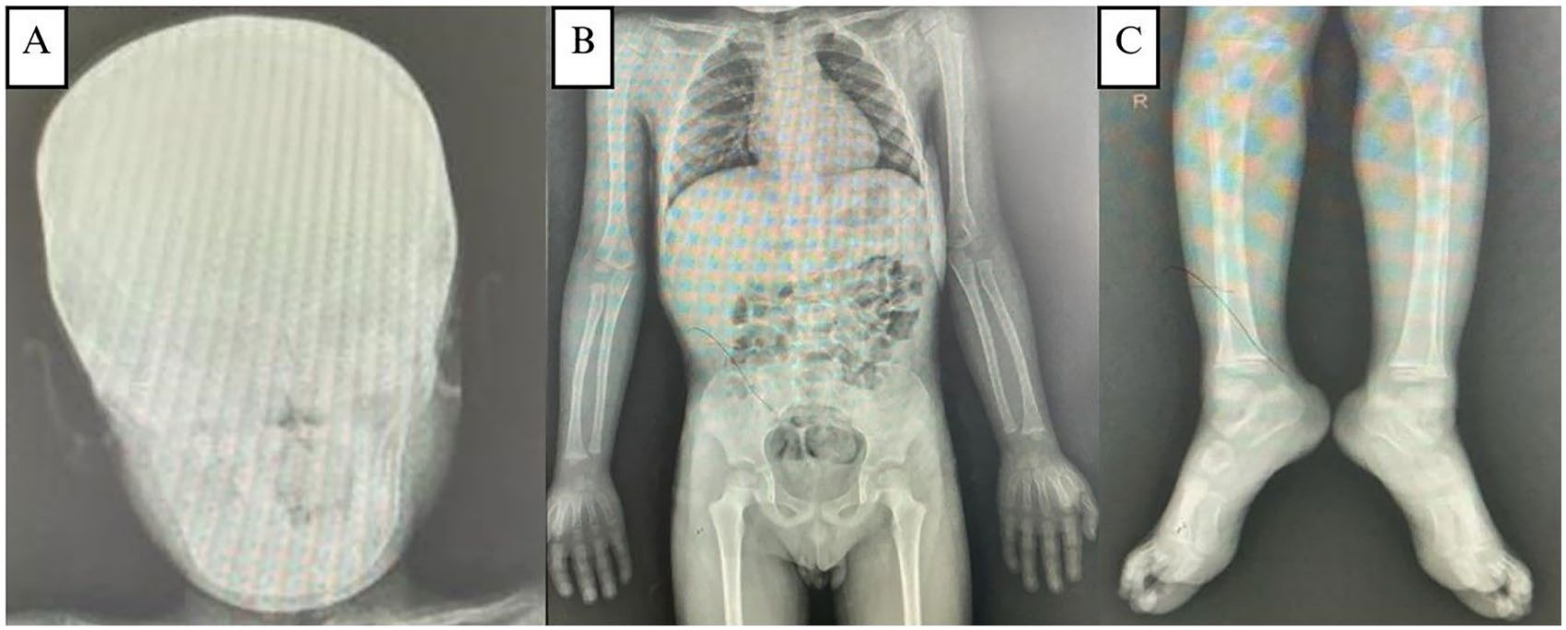
**(A, B, and C)** X-ray of the skull reveals calcification along with good bone trabeculation, inhomogeneous opacity in the mandibular area, an amorphous form, and distinct borders with regular edges. Both the pedicles and the corpus are intact. There are no apparent osteolytic or osteosclerotic lesions. An X-ray of the lower, upper, and pelvic extremities shows that there is strong trabeculation and bone structure. There is no apparent osteolysis or osteosclerosis.

Extraoral examination showed a mild facial asymmetry with a well-defined enlargement measuring 5 to 6 cm on the right side of the mandible. There were no mouth opening restrictions or swollen submandibular lymph nodes. Intraoral inspection displayed expansion of the buccal cortical plate, extending from the mandibular right first molar to the retromolar area (Figure 3(D)). Palpation revealed that it was hard and solid in consistency, showing no symptoms of paresthesia or mucosal inflammation. An OPG revealed a radiolucent, amorphous lesion in the right mandible associated with multiple unerupted permanent teeth (Figure 3(E)). CT imaging showed an expansile hard-tissue mass with cystic components measuring 37.3 × 45.7 mm and well-defined margins (Figure 3(F and G)). A skeletal survey demonstrated normal bone trabeculation without osteolytic or osteosclerotic changes (Figure 4(A–C)).

**Figure 5.**
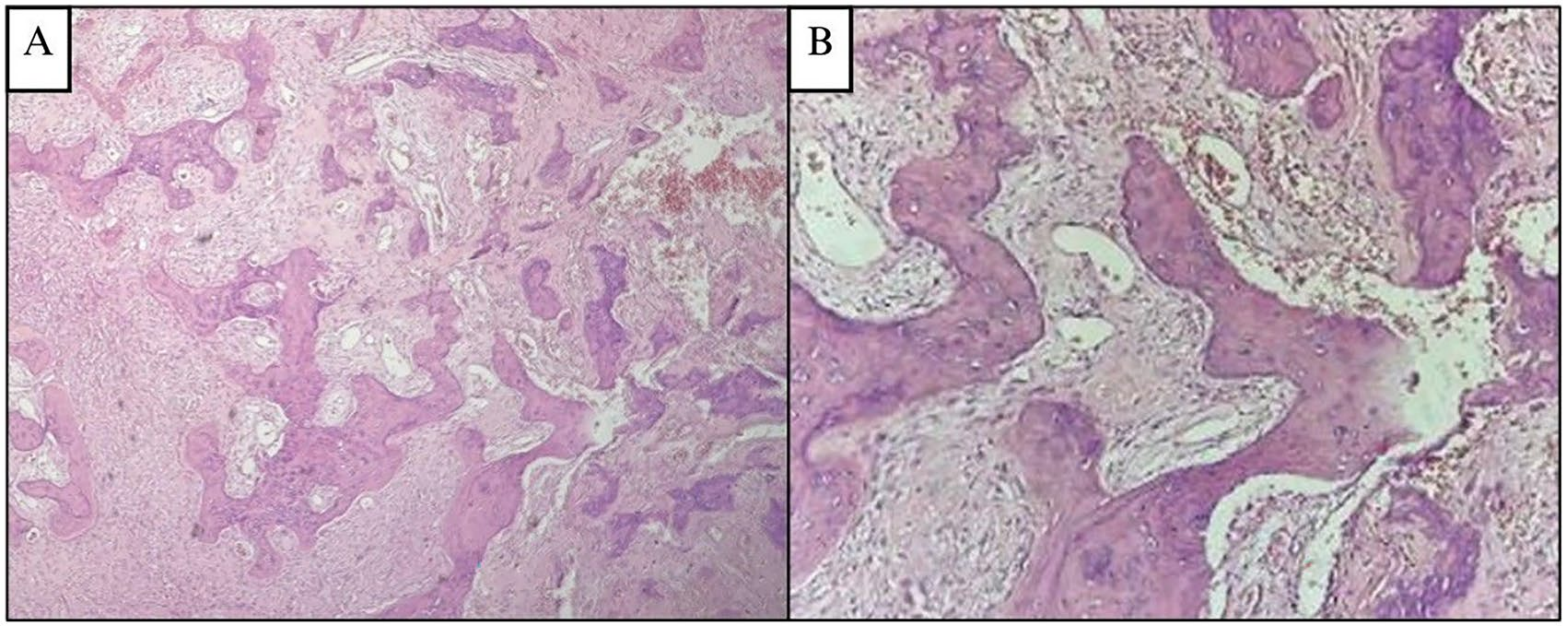
**(A and B) Case 2**, H&E sections at 4× and 20× magnification, showing an irregular trabecular bone tissue forming the letters ‘C’ and ‘S’ resembling a ‘Chinese letter’. The trabecular bone is mostly without osteoblastic rimming, and no osteoclasts are found. The fibrous connective tissue stroma has spindle-shaped fibroblast-like cells, is dense, and without atypia. Mitosis is very difficult to find. There were no signs of malignancy.

The patient underwent contour excision of the right mandible using a conservative approach with fissure and carbide Fraser burs to remove and remodel the affected bone (Figure 3(H)). The bone was softer than normal, facilitating reshaping. After saline irrigation and layered closure (Figure 3(I)), the patient recovered without complications and achieved satisfactory aesthetic results (Figure 3(C)). Histopathological examination showed irregular trabecular bone without osteoblastic rimming in a dense fibrous stroma lacking atypia or malignancy (Figure 5(A and B)). A provisional diagnosis of fibrous dysplasia was made, with ameloblastoma and odontogenic myxoma considered as differential diagnosis.

### Case 3

#### Subjective and objective examination

**Figure 6.**
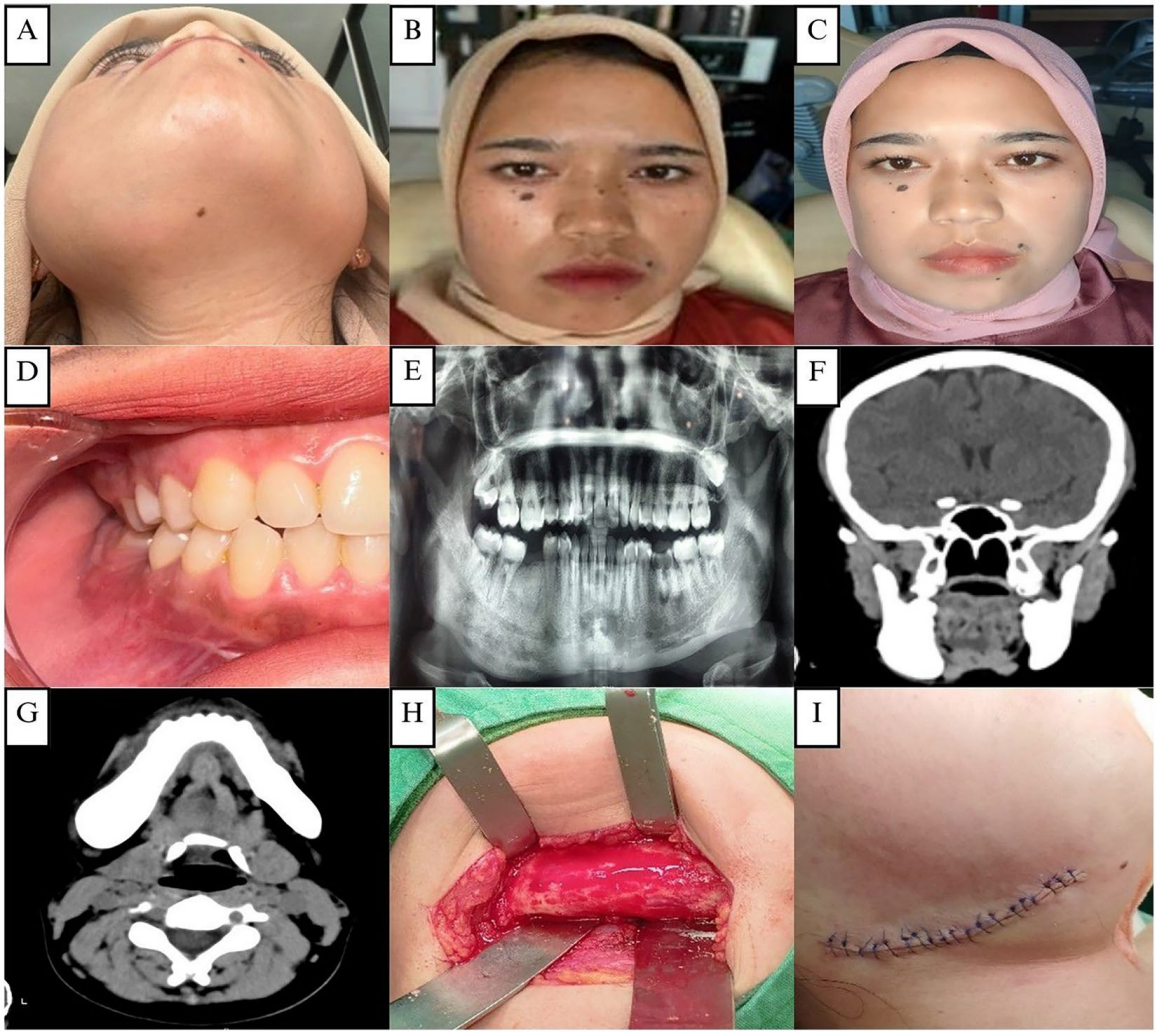
**(A and B)** The third case, a 23-year-old adult female patient with swelling in the right mandibular area. **(C)** Two weeks following the patient’s operation. **(D)** Pre-operative intraoral clinical appearance **(E)**. OPG showing an enlargement in the right ramus mandibular area with the typical ‘ground glass’ like opacification of the bone, which blended with the adjacent normal bone. **(F–G)** CT scan showing the size of the right mandibular body is larger than the left, no bone erosion, and periosteal reaction is seen. **(H–I)** Surgical exposure, bone reshaping, and layers of closure of the right mandibular intraoral area.

A 23-year-old adult female with a chief complaint of asymptomatic swelling in the right mandible came to Oral and Maxillofacial Clinics, RSUD Temanggung (Figure 6(A and B)). The swelling expansion was slow, gradually increasing during the last 2 years. There was no history of any trauma to the region and no pain or paresthesia in the affected area. Patient had a history of incisional biopsy in March 2024 with diagnosis of fibrous dysplasia. There was no medical history of allergies.

On physical examination, the bony expansion seemed to involve the buccal cortex and the inferior borders of the mandibles in the 45–48 regions (Figure 6(D)). When palpated, it was found to be solid and hard, with no signs of mucosal irritation or paresthesia. Odontogenic tumours or cysts, fibro-osseous tumors of the jaw, such as FD or ossifying fibromatoma, osteoblastomas, were among the differential diagnoses.

Orthopantomograms (Figure 6(E)) showed multiple tooth impactions, multiple pulp necrosis, intact mandibular bone system, and enlargement in the right ramus mandibular area with the typical ‘ground glass’ like opacification of the bone which blended with the adjacent normal bone. CT scan showed the size of the right mandibular body is larger than the left, no bone erosion and periosteal reaction is seen. No intracerebral or cerebelli abnormalities were seen (Figure 6(F and G)).

**Figure 7.**
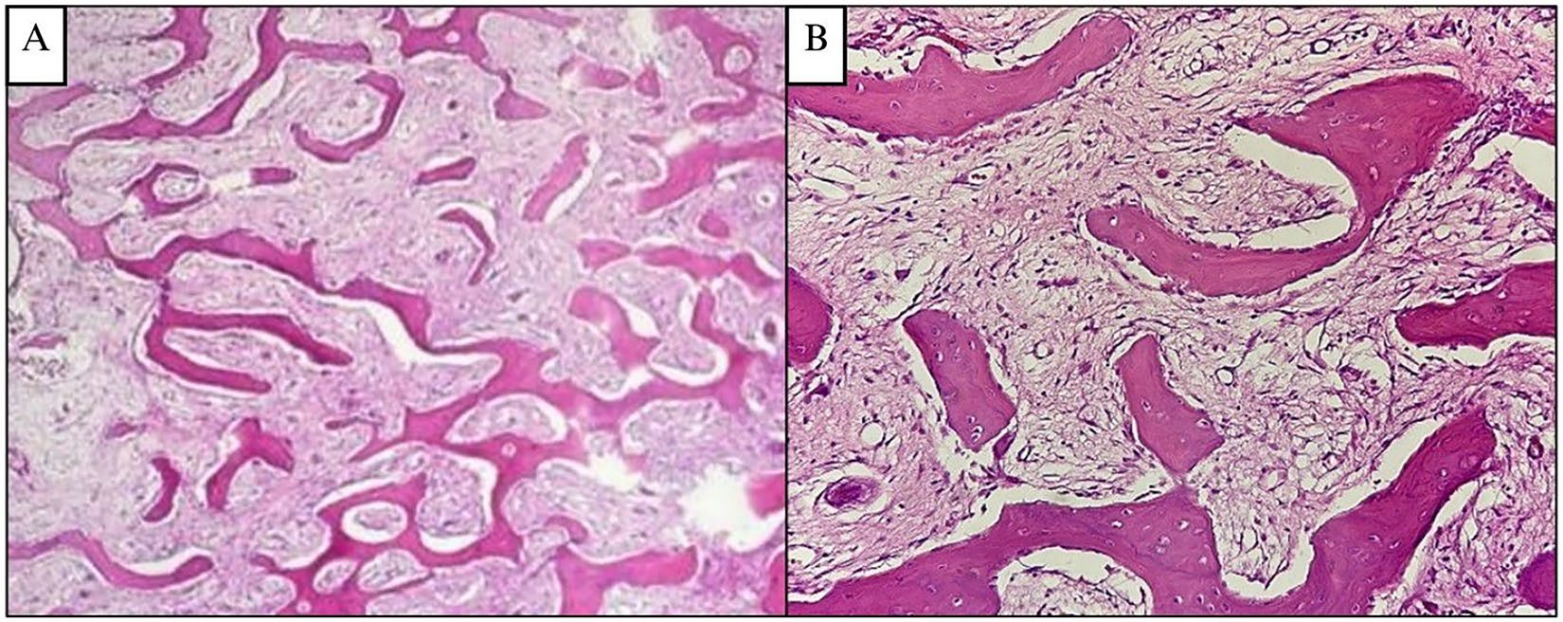
**(A and B) Case 3.** H&E sections magnified at 4× and 20× display irregular trabecular bone tissue that branches and anastomoses to create the letters ‘C’ and ‘S’, which have a Chinese letter-like appearance. The trabecular bone is mostly without osteoblastic rimming, and osteoclasts were not found. The thick, atypia-free fibrous connective tissue stroma contains spindle-shaped fibroblast-like cells.

The patient underwent surgical excision and bone recontouring under general anesthesia. Using an extraoral submandibular approach, excess bone was removed with a long fissure bur, followed by trimming and contouring with a carbide Fraser bur. The bone was softer, browner, and more granular than normal, with moderate bleeding after osteotomy (Figure 6(H)). The surgical site was closed in layers with 3/0 polysorb silk and a pressure dressing applied (Figure 6(I)). Postoperatively, the patient had no complaint at the 2-week follow-up and was satisfied with the aesthetic outcome (Figure 6(C)). The histopathological examination of the tumor showed irregular trabecular bone tissue. The fibrous connective tissue stroma has spindle-shaped fibroblast-like cells, dense, and without atypia (Figure 7(A and B)). A provisional diagnosis of fibrous dysplasia of the mandible was made.

### Case 4

#### Subjective and objective examination

A 15-year-old adult female with the chief complaint of asymptomatic enlargement in the mandible area referred to the Oral and Maxillofacial Clinics, Dr. Sardjito Hospital Yogyakarta. During the previous three years, the swelling growth was moderate and increased slowly. A biopsy was carried out at Temanggung Regional Hospital in August 2021, with the results of fibrous dysplasia. The patient denies weight loss, no complaint of swallowing pain, and no history of drug allergies or systemic diseases.

**Figure 8.**
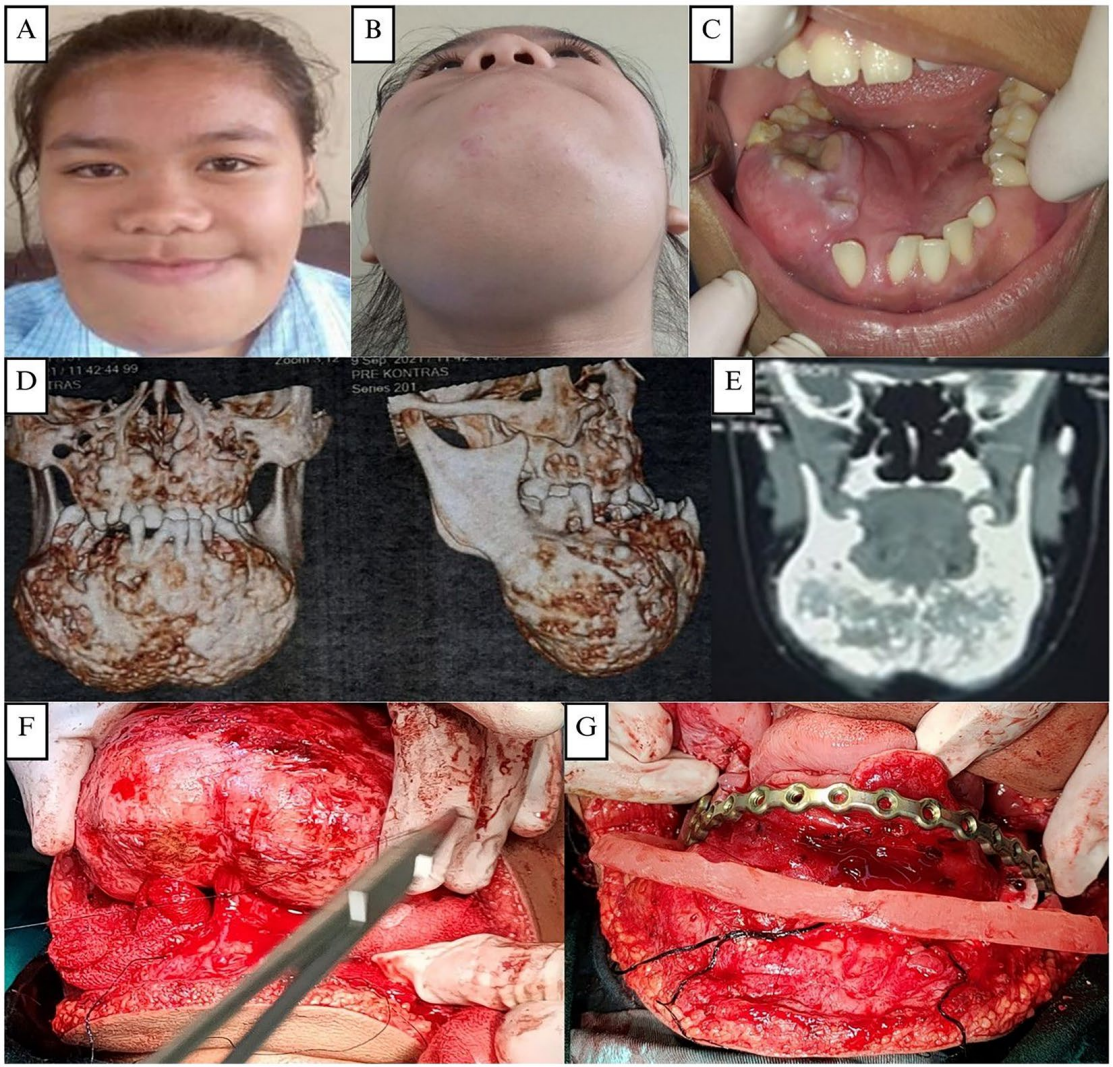
**(A and B)** The fourth case, a 15-year-old adult female patient with swelling in the right mandibular area. **(C)** Pre-operative intraoral clinical appearance. **(D–E)** CT scan showing an enlargement in the right ramus mandibular area with the typical ‘ground glass’ like opacification of the lesional bone, which blended with the adjacent normal bone. CT scan showing the size of the right mandibular body is larger than the left, no bone erosion and periosteal reaction is seen. **(F–G)** Surgical exposure, bone reshaping, and layers of closure of the right mandibular intraoral area.

**Figure 9.**
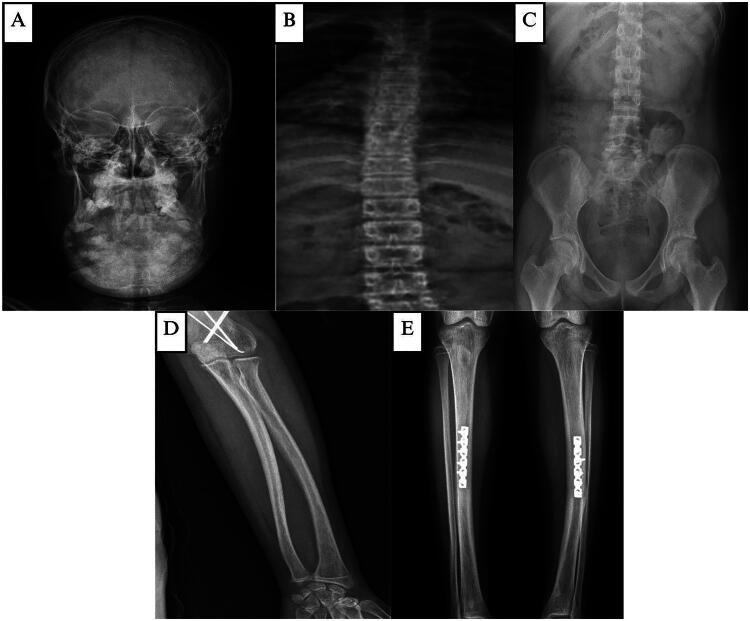
**(A)** Skeletal survey of the fourth patient, X-ray of the head shows good bone trabeculation, inhomogeneous opacity in the mandibular region, amorphous shape, clear borders with regular edges, accompanied by calcification. **(B–E)** X-ray of the vertebrae, pelvic, upper and lower extremity shows good bone structure and trabeculation. No osteolytic or osteosclerotic bone are visible.

Extraoral examination revealed facial asymmetry with a firm, non-tender mandibular mass extending from the left to the right corpus and bilateral maxillary enlargement, without skin changes or palpable lymph nodes (Figure 8(A and B)). Intraorally, a hard, non-mobile mass was noted in the left mandibular body extending to the 47–37 region, with normal mucosal color, dental crowding, and unerupted teeth (Figure 8(C)). CT imaging demonstrated solid, calcified expansile masses in the mandible (9.8 × 6.5 × 6.0 cm) and maxilla, without cortical destruction or soft-tissue invasion (Figure 8(D and E)). Skeletal survey radiographs showed good bone structure and trabeculation with inhomogeneous mandibular opacity and calcification, and no osteolysis or osteosclerosis in the axial or appendicular skeleton (Figure 9(A–E)).

**Figure 10.**
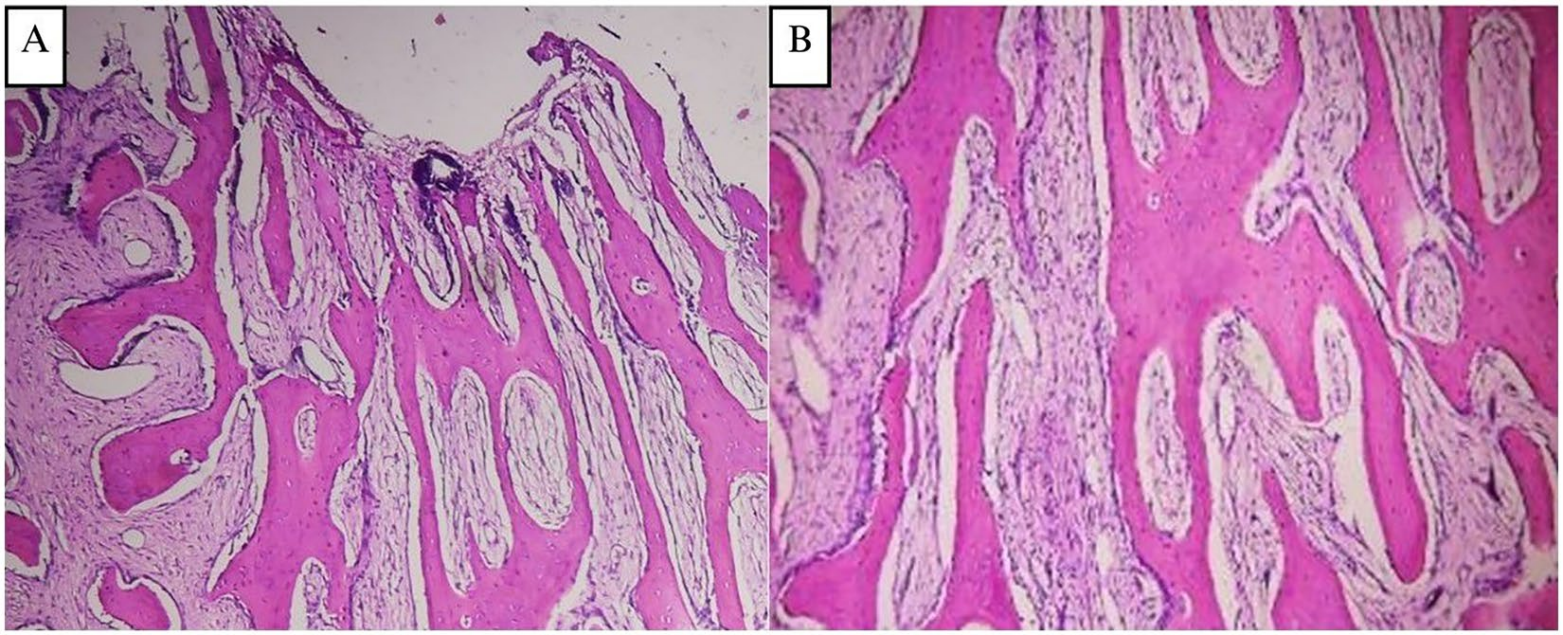
**(A and B) Case 4.** The irregular trabecular bone tissue shown in H&E sections amplified at 4× and 20× branches and anastomoses to form the letters ‘C’ and ‘S’, like a Chinese letter. Osteoblastic rimming is absent from the trabecular bone, and osteoclasts were not found. Fibroblast-like cells in the form of spindles are present in the thick, atypia-free fibrous connective tissue stroma. Finding mitosis is a quite hard.

The patient was planned for staged surgical excision of the fibrous dysplasia starting from the mandibula, and continued with the maxilla later on. The patient underwent a lower jaw segmental resection *via* a combined intraoral and extraoral approach with immediate reconstruction using 19- holes titanium reconstruction plate in November 2021 (Figure 8(F and G)). Histopathological findings of the mandibular tumor were consistent with fibrous dysplasia (Figure 10(A and B)). Postoperatively, the patient did well without complications.

**Figure 11.**
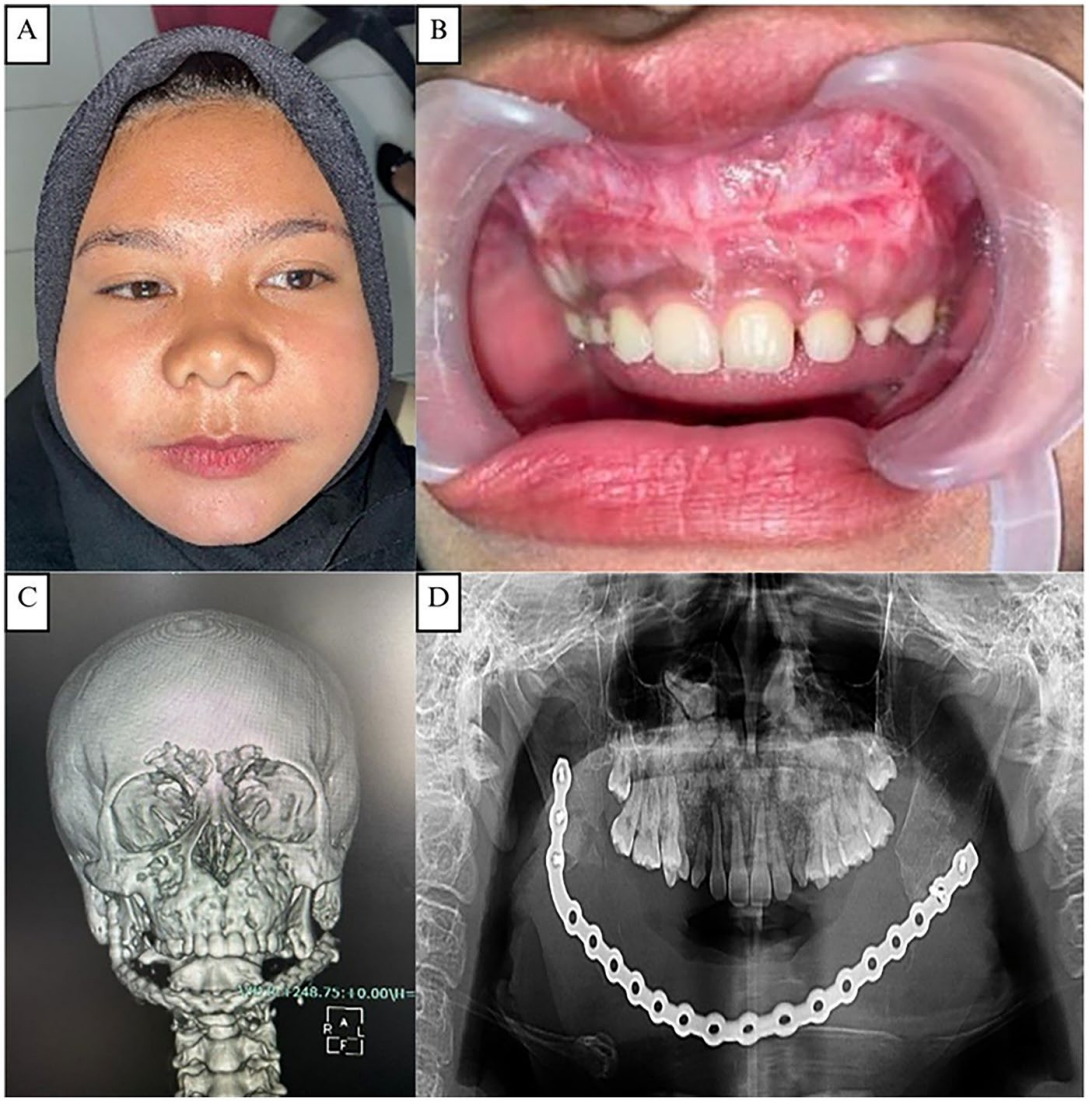
**(A)** The fourth case, a patient after 2 years follow up complaint with swelling in the maxillary area. **(B)** Pre-operative intraoral clinical appearance. **(C)** The CT scan showed a solid mass accompanied by calcification in the maxillary bones without soft tissue infiltration. **(D)** OPG showing an internal fixation installed with a bridging plate and screws, which fixed the mandibular corpus defect.

The patient presented on March 2024 for another surgery for the maxillary fibrous dysplasia. The patient does not feel pain or soreness. Extraoral examination revealed a minimally asymmetrical face, with a lump on the maxilla extending from the left to the right side (Figure 11(A)). There is no pressure pain, the texture tends to be hard, and in some areas it feels softer. Intraoral examination revealed a shallowing of the vestibule in the maxillary area was seen. The posterior teeth appear to be slightly covered by soft tissue (Figure 11(B)). CT scan showed similar findings from the previous examinations prior to the first surgery (Figure 11(C)). OPG showing an internal fixation installed with a bridging plate and screws, which fixed the mandibular corpus defect (Figure 11(D)).

The patient underwent surgical exicision and bone recontouring using a conservative approach with fissure and carbide Fraser burs to restore facial symmetry. The softer bone facilitated reshaping, and the site was irrigated and closed in layers. The patient recovered uneventfully with an excellent aesthetic outcome. Histopathological findings were consistent of fibrous dysplasia.

#### Surgical technique

An intraoral approach with maxillary contour excision was planned to restore the patient’s facial symmetry and aesthetics. A conservative surgical procedure was performed using a long fissure bur and a carbide Fraser bur to remove some bone and restore the deformed area. The bone’s consistency was softer than usual, which made it easier to trim and shave. The surgical sites were carefully irrigated with normal saline before layer-wise closure. Postoperatively, the patient did well without complications. The surgical outcome was excellent in terms of appearance and the recovery process following the procedure proceeded well.

## Discussion

Fibrous dysplasia is an uncommon skeletal anomaly that is characterized by a halted maturation at the woven bone stage [6]. It affects both sexes equally and accounts for 2.5% of bone tumor and 7% of benign bone tumours [11]. This tumor may occur at any stage of childhood or adulthood, but most cases are identified by the time an individual reaches the age of 30 [12]. According to some authors, the tumor tends to stop around puberty. Other research suggests that it continues into adulthood [13,14]. Hart et al. reported that FD was present before the age of 15, regardless of site [15]. In our study, 3 patients showed FD occuring in different groups of age.

The most common complaints when FD occurs in the facial skeleton’s bones are asymmetry and oedema [16]. Other symptoms such as malocclusion, facial distortion, orbital dystopia, sinusitis, and nasal dysfunction also reported [6,16]. Approximately 36.3% of fibrous dysplasia cases are challenging to diagnose due to the absence of distinctive clinical features, while 63.6% of patients present with non-specific symptoms such as pain or swelling [10]. The maxilla is more commonly affected by FD than the mandible [10]. However, in our case series, only the first patient presented maxillary FD. The second and the third patient presented mandibular FD, while the fourth patient presented this tumor both in the maxilla and mandible.

Cone-beam computed tomography is the preferred imaging test because it provides a more comprehensive view of the location and size of the tumor, which aids in surgical procedure planning. The ‘ground glass’ look, which has a fine cortex and no clearly defined borders, is the most common X-ray characteristic of craniofacial FD [17]. As the tumor progresses, it may result in abnormalities and aesthetic issues. In the present cases, the clinical, radiographic, and histopathological features were consistent with fibrous dysplasia, allowing a confident diagnosis without the need for additional genetic testing, and surgical management was planned accordingly. None of the four patients underwent genetic testing or a syndromic association (e.g. McCune-Albright syndrome), thus, there was no evidence regarding syndromic association.

The main purpose of the treatment is the correction of the functionality in combination with aesthetic effects. Treatment with surgical removal of the afflicted bone tissue is typically successful. However, it causes severe functional and cosmetic deficiencies in addition to chronic surgical consequences [18,19]. It is also crucial to remember that, except from cases in which they are completely removed, 20–25% of these tumors grow back following therapy [17]. This makes it impossible to predict whether or not FD will recur. Less than 1% of FD cases have been reported to have malignant transformation to be a sarcoma, most often osteosarcoma, but fibrosarcoma, chondrosarcoma, and malignant fibro-histiocytoma have also been reported [9,20].

Based on current clinical guidelines and contemporary reviews on craniofacial fibrous dysplasia, the decision between reshaping/debulking and segmental excision needs a study of the tumor’s biological behavior, functional impact, and pace of progression. For quiescent or non-aggressive tumors that stay stable over time, reshaping or debulking is typically advised, especially when the major concerns are cosmetic deformity or moderate asymmetry without discomfort, neurological damage, or functional disturbance. On the other hand, when the disease exhibits aggressive features like rapid enlargement, cortical erosion, severe pain, or compression of important structures, or when it results in functional impairment like malocclusion, visual disturbance, airway obstruction, or recurrent deformity following multiple contouring procedures, resection – often with vascularized bone flap reconstruction – is recommended [20,21].

The long-term effects and natural history of FD across all age groups have not been well studied. Standardized therapeutic procedures that consider age-specific factors such as bone maturity, growth, and skeletal development do not currently exist. Studies focusing on long-term results and treatment approaches unique to this age range might be lacking. In our case series, average follow-up period of the patients were 1 month and there were no signs of recurrence during those 1 month follow up. Long term follow-up is necessary to monitor recurrence in these patients.

## Conclusion

Fibrous dysplasia of the jaws remains a diagnostic challenge due to its overlapping clinical, radiographic, and histopathological features with other fibro-osseous lesions. In the present case series, the diagnosis was established through a combination of clinical evaluation, imaging findings, and histopathological confirmation. Surgical excision and bone recontouring provided satisfactory functional and aesthetic outcomes in most patients, while resective surgery with reconstruction was required in extensive disease. These findings support an individualized treatment strategy based on lesion extent, functional impairment, and aesthetic considerations, with long-term follow-up recommended to monitor potential recurrence.

## Data Availability

The data presented in this case report are available on-request to the corresponding author.
